# Research participant perceptions of personal utility in disclosure of individual research results from genomic analysis

**DOI:** 10.1007/s12687-024-00734-7

**Published:** 2024-09-18

**Authors:** Brenda Bogaert, Marie-Josée Crevier, Cindy Roth, Ralf J. Jox, Gaia Barazzetti

**Affiliations:** 1 Institut des humanités en médecine, Lausanne University Hospital and University of Lausanne, Lausanne, Switzerland; 2 Unité de consentement à la recherche, Lausanne University Hospital and University of Lausanne, Lausanne, Switzerland; 3 Gaia Barazzetti, EPFL, Lausanne, Switzerland

**Keywords:** Genomic return, Personal utility, Patient engagement, Sudden cardiac death

## Abstract

This article elaborates research participant perspectives on the communication of individual research results from genomic analyses. While most analyses focus on how to communicate results from the perspectives of clinicians or researchers, there is insufficient data on user perspectives and how this information may be used, valued, and interpreted by patients and their families. The concept of personal utility, which considers factors related to quality of life, including on how information may impact the person’s future decisions, has been shown to be particularly relevant to understand research participant perspectives and to move beyond clinical and analytic utility factors such as mortality and morbidity. This article draws from qualitative research of research participants awaiting genomic results in the case of sudden cardiac death. Our results show perspectives of personal utility in communication of genomic results, including cognitive, behavioral, and affective outcomes. Cognitive outcomes include gain of information, improved knowledge of etiology and inheritance characteristics, and curiosity for what might be found. Behavioral outcomes include being able to plan life decisions, while affective outcomes include various coping strategies used. We will also discuss the value of knowing negative results and incidental findings from the research participant’s perspective. This contribution gives suggestions on best practices to guide genome analysis returns, including incorporating participant wishes on individualized communication at the consent stage; developing relational autonomy approaches; and engaging them throughout the research trajectory.

**Supplementary Information:**

The online version contains supplementary material available at 10.1007/s12687-024-00734-7.

## Introduction

Progress in genomic research goes hand in hand with the assessment of the clinical value of genome analysis results and with their communication to research participants. Over the past decade, researchers, ELSI experts and policymakers have thoroughly considered these issues with a view to develop frameworks and guidelines for the disclosure of individual results in genomic research (Bookman 2006; Ravitsky and Wilfond 2006; Wolf et al. 2008; Dressler 2009; Hall et al. 2013). While discussion on “what” kind of results should be returned, “to whom”, “when” and “how” continues at an academic and policy level, there is a growing consensus on the general criteria that should be taken into account to assess the expected benefits and risks of returning individual results to genome research participants. These criteria include analytic and clinical validity, and the clinical utility or actionability of the results (Bookman 2006; Wolf et al. 2008; Fabsitz et al. 2010; Wolf et al. 2015). Current guidelines have been principally drafted based upon recommendations by clinicians, researchers, and scholars; however, the patient’s perspective has thus far been insufficiently developed and integrated into research and practice (Daack-Hirsch et al. 2013). At the current time, there is a considerable ambivalence and uncertainty in beliefs, norms, and actions stemming from differing expectations, interests, demands and tensions depending on different stakeholders and their values/interests (Kuiper et al. 2023a, Kupier et al. 2023b).

Researchers and clinicians are increasingly aware that analysing only the impact that variations and mutations may have on morbidity and mortality do not show the full picture. They are increasingly interested in understanding how the communication of such results may impact well-being. To do so, we need a better understanding of the ways in which patients and caregivers understand, appropriate and use this information (Barazzetti et al. 2014; Geelen et al. 2011). Research participant perspectives of what information will be pertinent to them and how they see, value, and plan to use this information merits further research. Previous quantitative and qualitative research shows that researchers, clinicians, patients and families all value the importance of communicating genetic and genomic findings (Mwaka et al. 2021; Char et al. 2018; Sanderson et al. 2017), although there remains considerable debate over which information is relevant, in particular the need to communicate (or not) negative results and incidental findings. Best practices on the communication of genomic results are therefore needed to inform not only clinical and research practice but also to guide policy and legislative reform (Thorogood et al. 2019).

The concept of personal utility, as a broad category of outcomes that include subjective, non-health related uses, is relevant to elucidate research participant perspectives on return of genomic results. Personal utility provides a broader notion of utility compared to analytic or clinical utility perspectives primarily focused on medical and research priorities (Kohler et al. 2017). While there is currently no consensus on the definition of personal utility, popular definitions in the context of genetics and genomics emphasize how this information may impact the person’s actions and decisions (Bunnick et al. 2015) and perspectives on how information may impact family planning, anticipation, and social assistance (Bale and Mitchell 2019). In this paper, we will use Kohler et al’s (2017) conceptualization of personal utility, which uses four outcomes: cognitive, behavioral, affective, and social outcomes. Table 1 summarizes the three outcomes we will discuss in line with our research results.

**Table 1:** Kohler’s cognitive, behavioral, and affective outcomes

Cognitive	Behavioral	Affective
Gain of information and its value to the person	The practical uses of genetic information, including capabilities for future social planning	Coping with health risks associated with one’s condition
Improved knowledge of the condition including etiology (cause or origin) and inheritance characteristics	Reproductive autonomy, including the decision to plan for children with a genetic risk	Feeling more in control of oneself and the situation
Self-knowledge, including possible identity changes that may come from having information on a genetic condition	Communication with relatives as a result of genetic information (due to possibilities of other family members as carriers)	Mental preparation for oneself and one’s family to visualize the future (such as reproductive planning)
Curiosity for what might be found and what it might mean		

As we can see from this table, there are three outcomes that are particularly relevant to understand research return. This includes a cognitive component, including increased knowledge of one’s condition; behavioral outcomes, such as using information gained to make reproductive choices or to communicate with family members; and finally affective outcomes, such as being better able to cope with health risks or feeling more in control. As we can see from the above list, these outcomes are interconnected: with increased knowledge, the person is better able to make practical choices about their lives and future and is likely to feel more in control and know better how to cope. This list may therefore be seen as a global perspective of how the individual person sees themselves, their relationships with family members, and their future in light of results return. From this perspective, it is quite different from a clinical outcome which is focused principally on treatment issues.

Our research investigates research participant perspectives on the communication of genomic results in the case of sudden cardiac death (SCD). The condition refers to an unforeseen, unexpected heart function loss, with the cause of death being either cessation of heart or irregular heart rhythm (Primorac et al. 2021). It may occur after a history of acquired cardiac disease but may also be the first manifestation of a genetic heart disease (Primorac et al. 2021). Given the devasting impact of SCD on patients and families, increasing knowledge about the molecular mechanisms and genetic drivers of this condition is vital to enhance risk stratification and personalize patient care (Isbister and Semsarian 2019; Fellman et al. 2020; Fellman et al. 2019). Testing relies on the clinical diagnosis of a cardiac genetic disorder based upon incidental findings or symptoms in an individual or family. Genomic testing helps to determine genetic susceptibility to SCD by identifying rare variants and high polygenic risk scores (Primorac et al. 2021). From a clinical perspective, genomic testing assists the clinician and the patient to consider risk factors and potential preventive or therapeutic interventions. However, the personal utility of receiving these results from their perspective has not (yet) been explored, including wishes about receiving negative results and incidental findings. The purpose of our study is help move forward the overall debate on personal utility and research communication from the research participant’s perspective for those who face a possible diagnosis of SCD, but in which there was not necessarily a sudden cardiac death (SCD) in the family nor the relevant genetic variant in an SCD-related gene identified. As will be discussed, the results may also help guide communication of genomic findings for this condition, although the purpose of the study was first and foremost to better understand the perspective of research participants, and in particular to explore the ethical questions around consent of communication of results.

## Materials and methods

The research project aimed to explore and document research participants’ perceptions on the communication of genome analysis results. It also had a practical aim: to improve guidelines and practice on the communication of genetic and genomic information in clinical research at the Vaud research hospital (CHUV) in Lausanne, Switzerland. A multidisciplinary group within the CHUV proposed a guide to communicate genetic findings to research participants; however, there is a need to better understand their perspectives to make them useful/relevant.

### Research context

While proposed as an independent project, this research project was coordinated with a larger research genomic research project (COPRAC) on sudden cardiac death at the Unit of Precision Medicine at Lausanne University Hospital (CHUV). The clinical study used WGS to investigate rare, deleterious mutations associated with cardiomyopathies or channelopathies in individuals suspected to suffer from hereditary forms, due to their family history or to suggestive clinical signs. The COPRAC study included 150 research participants with a family history of sudden cardiac death and/or a suspected genetic origin to an already diagnosed cardiac illness.

Genetic testing was performed in a clinically-accredited laboratory (ISO 15189:2013 certification). Variants were annotated and prioritized based on population and cohort allelic frequencies, predicted gene impact, and known association to disease. To further assess the pathogenicity of the top candidate variants, an expert group was formed including a medical geneticist, a genetic counsellor, a biologist and a bioinformatician. Whole genome sequencing was used for all individuals. However, only variants in genes previously associated with a cardiac phenotype (in silico gene panel) were analyzed to minimize the risk of incidental findings. Because the identification of such mutations would be of high clinical relevance, when such results have been obtained and validated, they were returned to the clinical study participants according to the CHUV hospital guidelines for returning results.

The participants in the clinical study were recruited among patients in the CHUV hospital cardiology department who gave their broad consent to the use of their samples and data for future research, including genomic research, and agreed to be re-contacted if clinically relevant results were to be found. An additional, specific consent was used to propose to these patients to participate in the clinical study which included specific information about the potential disclosure of genomic findings to participants in case clinically relevant variants were identified.

A multidisciplinary clinical consultation was offered to patients re-contacted for a return of results, involving cardiologists, medical geneticists and genetic counsellors to ensure optimal communication and care for the patients and their families. This consultation was put in place as standard clinical service in agreement with the clinical specialties concerned at CHUV (i.e., medical genetics and cardiology). However, the consultation was not part of the clinical study, nor of our qualitative research. The research to be discussed in this article took place when the person came to give their blood samples to participate in the COPRAC clinical study. As shown in Table 2 (the next section), all participants had a suspected genetic cause for their condition; however, not all had a clearly identified family history of SCD. It is to be noted that as our study was separate to the COPRAC study, we were not authorized by the ethics review board to have access to the clinical data as this is considered personal sensitive data that is not accessible to researchers conducting a qualitive study on patient’s perceptions according to the Swiss Human Research Act and the Swiss Federal Data Protection Act. Therefore, the data gathered reflects the perspectives and knowledge of research participants, which was sufficient for our research aim.

In the context of the study, it was decided by the research team to contact only those research participants whose specific genetic mutation was found. In practice, this meant that those whose specific genetic risk factor was not found were not recontacted. Recommendations developed by the Swiss Personalised Health Network in 2020 encourage researchers to communicate all medically relevant genetic findings to research participants and to exclude from participation those who do not want to receive this information (Blasimme et al. 2020). However, at the current time, there is no specific guidance on what to do in the case of negative results and/or incidental findings. By “incidental findings” we mean WGS results that were not in the scope of the results targeted by the clinical study. The clinical study did not intend to seek for these secondary findings, and it was made clear in the information to participants in the clinical study that only actionable results related to the variants targeted by the study would be returned. However, in our qualitative study, we have explored participants’ wishes regarding the return of such incidental findings. When we were asking the question, we were framing it in very general terms and referring to very general examples of variants related to hereditary cancer or late onset neurodegenerative diseases (based on examples provided in the survey by Middleton et al. 2016). However, we did not develop further on these examples, because we simply wanted to explore participants’ wishes regarding possible results outside the scope of the clinical study, and we wanted to avoid any misunderstanding by the participants in our interviews regarding the objective of the clinical study.

Participants who were included in the clinical study were informed about the qualitative study by collaborators of the Research Consent Unit of the CHUV. The information was given during their visit at the CHUV for blood sampling dedicated to the clinical study. The collaborators were trained to give this information and followed a pre-established plan drawn up by the principal investigator, outlining the main objectives of the study as well as the organizational and administrative aspects of the project. Persons interested in the study were given the opportunity to contact the principal investigator for any further information or questions before agreeing to participate. Those who were approached were offered time to think about their participation in the qualitative study, or to sign the written consent form on the day of the information. The interview was scheduled at a later date.

Data collection took place between 2019 and 2020. The study received ethical approval from the Cantonal ethics committee with the agreement that the service of medical genetics would be involved in the assessment of the clinical utility of research results and in the communication of results to the research participants involved.

### Study design

The study was qualitative, consisting of semi-structured interviews with research participants while they were awaiting their test results. The interview guide was developed based upon a literature review and discussions with the qualitative research team, consisting of GB and MC (the guide is in a supplementary file). The interviews were conducted by two researchers (GB and MC) based upon three main questions with additional probes for each question. GB has experience in qualitative research on ethical and social issues related to genomics.

### Participant selection

The inclusion criteria for our study included: (1) adult men and women aged 20–80; (2) participants who were awaiting their results in the COPRAC study; (3) willingness to participate in the study on perceptions of personal utility. Exclusion criteria were insufficient knowledge of French, insufficient knowledge of their cardiac condition, and/or insufficient decision-making capacity. We approached participants who were already selected according to the COPRAC study inclusion/exclusion criteria. Participants were recruited and given information on the study by the research team when they came to the CHUV to give blood for the study. Participants signed a written consent form on the day of the blood test and the interview was scheduled at a later date.

Participants in the clinical study included patients of the CHUV hospital cardiology department suspected to suffer from hereditary forms, due to their family history or to suggestive clinical signs. Part of these patients had a previous history of genetic testing that did not confirm the pathogenic variant. In our qualitative study, we were able to recruit a mixture of patients with clinical history only in cardiology or in cardiology and genetics. We have considered the different disease-related context in our analysis of individual results. However, we were not able to infer any general conclusion regarding the disease-related context because of the small number of participants in our study. The table below specifies the disease-related context for each individual (to be noted that the information relates to the individual disease-related context before their participation in the clinical study). The research participants being interviewed were awaiting the test result for their condition. It is to be noted they faced considerable uncertainty in participation in the study. The study group was large: it included either those followed in cardiology and with a family history (suspicion but not confirmation of a genetic link) or those followed in cardiology and who had a first genetic test which did not give a clear result. This meant that while each participant had a history of cardiac problems and most had a family history of a cardiac condition, the family history itself was not necessarily clear or linked to SCD. In our interviews, none specifically discussed whether a family member was found to have a SCD risk allele in the family (via test or post-mortem sample from a close relative) or even knew the source of the cardiac condition in their family. Some had also already participated in other research studies, which also came out negative. Thus, while there was considerable hope that a result could be found to explain their condition by participating in the study, from their perspective, this was largely uncertain.

It is also to be noted that we did not intend to process personal sensitive data of the patients interviewed in the framework of our qualitative study. Moreover, the ethics authorization we obtained for our qualitative study did not include the permission to process such sensitive clinical data which should remain accessible only by the research and clinical staff involved in the clinical study. As our qualitative study was conducted in parallel to the clinical study, it was separate from the clinical study.

### Sample size and composition

Initially, 12 persons were contacted to participate in the study and finally nine persons participated. 7 interviews were conducted before the COVID crisis (November 2019 – February 2020) and 2 additional interviews during the crisis (August – September 2020). Eight men and one woman were interviewed from a variety of age groups. Table 2 summarizes the main characteristics, disease-related context, and family history. It is to be noted that this information is as described by the research participants themselves. While some participants had a good knowledge of their condition (including using medical terminology), others were unable to describe the current knowledge of their condition. This was not necessarily due to a lack of health literacy in all cases, as their healthcare journey involved a significant amount of uncertainty. Several noted in particular different clinician perspectives on whether the cause of their cardiac condition was likely to be genetic or due to another factor.

**Table 2:** Interviewee characteristics

Participant	Gender	Age range	Disease-related context (before the clinical study)	Known family history (Y/*N*)
P1	M	40–50	Cardiology	N
P2	M	60–70	Cardiology and genetics	Y
P3	M	60–70	Cardiology	Y
P4	M	20–30	Cardiology	Y
P5	M	50–60	Cardiology and genetics	Y
P6	F	60–70	Cardiology and genetics	Y
P7	M	20–30	Cardiology and genetics	N
P8	M	40–50	Cardiology	Y
P9	M	70–80	Cardiology	Y

### Data recording, duration, and anonymization

Interviews lasted from 30 to 70 min. Interviews were audio recorded with the consent of the participants and transcribed verbatim by a professional transcription service and were destroyed after the transcription. The interviews were pseudonymized (with a coding P1, P2, P3, etc.) and personal details, including names of persons (participant names, interviewee names, doctor names, etc.), were removed in the text of the transcriptions to prevent identification of participants. The transcriptions were not returned to the participants.

### Analysis

Following Braun and Clarke’s guidelines on conducting thematic analysis (2006), data analysis was conducted by GB and MC to identify codes from the data. They conducted an independent double code of the entire data and then came together to agree on the coding. Encoding disagreements were resolved by referring directly to raw data. The coding was input into MAXQVA software to help organize data and facilitate interdisciplinary exchange between researchers. An independent check of the coding was conducted by BB. The analysis followed both a deductive and inductive approach, as the concept of personal utility helped to analyze the data.

## Results

Our results highlight research participant perspectives on the communication of genomic results in the context of sudden cardiac death. We rely on the distinctions introduced by Kohler et al. (2017) to organize our results, which are organized according to cognitive, behavioral, and affective outcomes.

### Cognitive outcomes: the value of genomic information

Respondents confirmed that the communication of genomic results had cognitive value from their perspective. This also included knowing negative results and incidental findings. All participants interviewed (*n* = 9) indicated the value of having this information, in particular concerning etiology of their disease. Indeed, the uncertainty surrounding the origin of their condition was a source of strain, in particular when there was a suspected death in the family and/or when a genetic origin for their condition was suspected but not (yet) confirmed.

Our research results closely align with Kohler’s idea on self-knowledge and curiosity for what might be found and what it might mean. Indeed, participants indicated a curiosity in having access to all medically relevant information, both relating to and beyond the research in question. This curiosity included knowing both **negative results** (results that did not show the specific gene being studied) and **incidental findings** (potentially pathological findings not initially searched) for most of the patients interviewed (*n* = 8).

In the case of negative results, even this “non” information gave them the sense of gaining knowledge. For instance P7 says, “*It’s true that I would indeed like to have the results…so after if it is*,* let’s say*,* positive or negative*,* in both cases it’s good to have feedback on this result… even if I am told that there is nothing*,* well that…it’s always good to know*,* to have feedback.”* Likewise, P9 says, *“If there is nothing*,* there is nothing. Full stop. I’m not going to bang my head against the wall for this….but let me be told*,* let me be told…so we know that either it’s serious or we haven’t seen anything.”*

### Behavioral outcomes: planning life decisions

All persons interviewed (*n* = 9) stated that they would use the knowledge gained from the communication of research results to better plan their life decisions and those of their family members. Given the age of several of our respondents (and the fact that sudden cardiac death could mean sudden death), behavioral outcomes also related to being able to plan end of life care (and not just reproductive autonomy).

Research participants said that they did not necessarily understand the knowledge gained in terms of clinical utility (many had doubts that this information would be immediately actionable in terms of treatment plans, at least in the case of their cardiac disease), but it was related to their capabilities in their overall lives. Therefore, it is to be clarified that Kohler’s behavioral outcome stresses the capability to act on decisions, rather than whether or not these plans have been enacted upon. In other words, what is important is feeling and being able to act (agency), rather than the actual act itself. These behavioral outcomes included: (1) anticipating future medical care and other life decisions, including for their cardiac condition or for other diseases found in genomic analysis; (2) participating in future research; (3) making arrangements for life decisions and/or end of life planning. Table 3 summarizes these themes.

**Table 3:** Perspectives on knowing genomic results, incidental findings, and negative results

Theme	Citation
Anticipating future medical care and life decisions	P7: *“I’d rather know…before the day I find out it’s too late.”*
	*P2: “I am in a perspective of knowing things. I don’t want to be ignorant in the sense of not wanting to know.”*
	*P4: “it’s always good to know…I start from the principle that from the moment that we discover something*,* we must inform…because a little thing can hide a bigger one or even lots of little things. As long as something else is discovered*,* even if it has nothing to do*,* we must inform.”*
	*P5: “Yes*,* I would like to have the results whether positive or negative…if it’s negative*,* once again*,* I think it will be again in a few years that we can start something else again and then look elsewhere and then continue.”*
Making arrangements for life decisions and/or end-of-life planning	*P9: “We must know where we are going….it helps me make arrangements to avoid problems.”*
	*P2: “I’m 70*,* I feel like I lived well…it allows me to make arrangements quite early*,* to do things*,* to settle all my things and not to be caught off guard.”*

These behavioral outcomes also had a strong relational component. Although participants believed in the individual value of having their results returned, they were just as concerned of its value for others. Thus, P2 explains, *“genetics is something that is transmitted…it may be useful for me to know*,* it’s always good to know*,* but especially for my next generation or certainly the generation of my children or grandchildren.”* This long-term perspective of results for their families was an important motivation to participate in research.

### Affective outcomes: coping strategies

The third theme that emerged from the interviews was the possibility of using the results to be able to better “cope” with the realities of their condition. As P4 says, “*as long as I know of other pathologies*,* necessarily I can anticipate… I am aware of the thing*,* I can anticipate how to solve the problem and that is not what will prevent me from getting up in the morning*,* from not sleeping.”* As we can see from this quotation, P4 does not see this information as a blocking factor: indeed, it is a facilitating factor that will help him feel more in control of his life.

Two interviewees also discussed the role of stress and emotions on coping. For instance, P1 believed that emotions may (also) have been responsible for her condition and knowing this information may help her to manage it. PI says, *“I would be interested in already knowing why it happened and then how it could evolve…if it’s a little bit also related to emotions….for me personally*,* it would be important.”* Similarly, P3 says, *“if there is a remedy for this*,* if we can correct it*,* intervene or if it is a lifestyle to improve*,* if it is a behaviour to improve*,* if it is stress…if we can eventually find a solution to alleviate this problem*,* I think that’s good.”*

As we can see from these quotations (and closely tied to the previous two themes), research participants believed that they would be able to better cope if they had more knowledge of their condition, including aggravating risk factors such as stress. Indeed, many of the participants had faced long healthcare journeys, in which they had to live with uncertain diagnosis. Therefore, a positive result from their perspective (meaning “something” was found) would enable them to find strategies to cope.

## Discussion

Overall, our study contributes to better understanding research participants’ perceptions of communication of the results of genome analysis, including their expectations, needs, and implications for relatives. Our research also lends further support to previous data showing a strong desire by research participants to know incidental and/or opportunistic findings in the case of serious and/or medically actionable illnesses (DeWert et al. 2021; Yamamoto et al. 2017; Middleton et al. 2016; Wright et al. 2014; Christenhusz et al. 2013). However, our research also suggests that they may also be interested in those results that are not immediately actionable, including a desire to receive negative or “non results.”

Our study also helps expand understandings of personal utility in the context of genomics and genetics testing. In the first place, current definitions (Bunnik et al. 2015) are focused on an individual capacity to engage in actionable behaviors. However, as these interviews have shown, this desire to know corresponds to cognitive and affective outcomes, as it helped persons gain self-knowledge and feelings of control (even when it did not lead to any concrete behaviors per se). In other words, it was a question of gaining capabilities (the capability to act based upon increased self-knowledge) but not necessarily immediate functionings.

Our results also suggest a more relational concept of personal utility compared to previous definitions focused on the individual. Indeed, participants understood their value from a relational perspective (Urban and Schweda 2018), which included their families.

Even though all of the persons interviewed in our study wished to know their research results, we are not seeking to make a strong statement on whether researchers/healthcare providers should *necessarily* disclose results to them and their families. In the context of genomic testing, while the importance of respecting individual choices to know or not know is recognized (Thorogood et al. 2019), as results may also implicate families and as well present risk factors for the patients and others, there remains considerable debate on the duty to tell (Parker and Lucassen 2018; Pullman and Hodgkinson 2006), including in the case of SCD (Bak et al. 2018). It should be noted that in the research context under study, the guidance on returning actionable research results from the Swiss Personalized Health Network (SPHN) encourage that participants be excluded from genomic research studies if they refuse to have their results communicated (see Blasimme et al. 2020 and SPHN 2020 recommendation 2).

It was therefore to be expected that the participants interviewed wished to know their results. This study however helped better understand the “greyer” areas of incidental findings and negative test results, which do not (yet) have recommendations for the Swiss context.

The main conclusions of this research can be stated as follows: most research participants see a value both for themselves and their families in communication of genome analysis results, including the possibility to have access to negative results and incidental findings. This information was seen as a resource to help them, and their families, anticipate their future life decisions and gain control of their lives. However, this also implied an expectation that they should have the possibility to have access to information that could be relevant to them and their families, including negative information (even if not necessarily considered relevant to the healthcare plan).

Future research can investigate how to best incorporate research participant wishes on results communication at the consent stage, including investigating whether their involvement can help facilitate this process. We also suggest more studies to explore reasons for refusal to know given the strong relational perspective coming from our research participants toward research. Furthermore, as our research took place during the “waiting stage” of the project, we were also not able to explore research participant perspectives on how they perceived returns of variants of unknown significance (VUS). This subject raises many ethical concerns, including the potential harms from receiving this information (Pollard et al. 2019; Hofmann et al. 2016). This subject also merits further research to develop best practices for the clinical and research context.

## Limitations

We acknowledge that the methodology presented above has certain limitations. Even though interviewees represented a diversity of profiles, it is possible that some themes were not present due to the small number of interviews conducted. In addition, we acknowledge that genders were not equally represented in this research. However, while the number of interviews was slightly less than initially planned (12 vs. 9), the interdisciplinary research team has confidence in the saturation of data as despite age/gender differences, the themes that emerged closely correlate among participants from different demographics. In addition, while GB and MC conducted the interviews and the preliminary data analysis, the inclusion of an outside researcher (BB) enabled an independent review. This two-step process enables us to have high confidence in data saturation despite the small sample size. In addition, a recent systematic review (Hennink and Kaiser 2022) has confirmed that with 9 interviews, it is possible to achieve saturation. However, due to the small population of this study, we recommend research with a wider population to confirm these preliminary findings. It should also be noted that we did not have clinical data for this study due to legal restrictions on qualitative research in Switzerland.

## Conclusion

Our research brings further understandings of the value of genomic result return from the research participant perspective. Lessons can be learned from this research to better plan research consent, results communication and to foster/encourage patient engagement. Lessons learned are relevant for the Swiss, European, but also for a wider context, although they will need to be adapted to each individual country/situation. The following are those that can help guide clinical practice and research:

### Lesson 1: Exploring wishes on results return, including about incidental findings and negative results, can already be done at the consent stage

From the research participant perspective, communicating results – including incidental findings and negative results - had cognitive value to help them to better understand their illness, gain control, and anticipate life decisions. These findings suggest that options on results disclosure can be discussed with the person at the consent stage to better personalize what information they wish to know, including in what temporality and by what means.

### Lesson 2: It’s a “family affair”: relational autonomy approaches need to be incorporated into genomic research communication.

Our results showed that research participants did not conceive of personal utility only from the individual perspective, but rather from a family perspective, as genomic results inevitably affect their life decision planning and those of others. As most consent processes take an individual perspective, this means we are missing this relational approach.

### Lesson 3: Research participants wish to be engaged throughout the research trajectory

Our results showed that research participants wish to be involved in research, not only for themselves, but also for their families. From this perspective, following up with patients throughout the research trajectory remains important and includes communicating negative results and overall research findings, should they wish. To do this well, researchers and clinicians will need to develop tools that favor health literacy (Haga et al. 2014) and help train genetic counsellors and researchers in how to restitute results in an accessible way.

## Electronic supplementary material

Below is the link to the electronic supplementary material.

Supplementary Material 1

## Data Availability

Additional data are available from the corresponding author on reasonable request.
