# Supplementary material for: Research participant perceptions of personal utility in disclosure of individual research results from genomic analysis
Source: J Community Genet. 2024 Sep 18;15(5):529–38. doi: 10.1007/s12687-024-00734-7 (PMC11549069; doi:10.1007/s12687-024-00734-7)
Supplement: Supplementary file 1 — Supplementary Material 1 [file 12687_2024_734_MOESM1_ESM.docx]

**Additional Information for Reviewer/Editor**

**Dear Reviewer/Editor, as discussed in the point-by-point response, due to ethical and legal requirements, we had to limit the data provided in the text on family and personal history. We have therefore limited the column “Family and Personal History” in the text to only a Y/N to the question of whether there is a known family history. We are however providing additional information on family/personal history “for your eyes only” to help give additional information/context (to the extent that we are allowed to provide). As noted in the text, as we do not have access to the case file, this is data coming from the interviews. However, please do not publish this table if the article is accepted given confidentiality issues. In addition, we have given an option in the text for readers to contact us to have these details for research purposes if needed, as described in the point-by-point response.**

**Table 2: Interviewee characteristics**

| **Participant** | **Gender** | **Age range** | **Disease-related context (before the clinical study)** | **Family and Personal History (according to research participants)** |
| --- | --- | --- | --- | --- |
| **P1** | M | 40-50 | Cardiology | Suspicion of genetic origin but first person in family to take tests; family history is uncertain as mother/father do not suffer from cardiac problems, but brothers/children will potentially be tested in the future depending on results |
| **P2** | M | 60-70 | Cardiology and genetics | Suspicion of genetic origin; son also being followed but currently no evidence that he suffers from the same condition |
| **P3** | M | 60-70 | Cardiology | Suffers from cardiac problems but cause not yet identified; son appears to suffer from same condition according to research participant |
| **P4** | M | 20-30 | Cardiology | Previous history of cardiac problems; suspicion of genetic cause on mother’s side but unsure if tests were carried out |
| **P5** | M | 50-60 | Cardiology and genetics | Family history suggests genetic cause but has not yet been identified (previous personal history of cardiac problems, brother also suffers from cardiac problems) |
| **P6** | F | 60-70 | Cardiology and genetics | Family history of sudden cardiac death (first cousins and mother died) |
| **P7** | M | 20-30 | Cardiology and genetics | Previous history of cardiac arrest and genetic cause suspected but unconfirmed; research participant does not have children and family history does not indicate cardiac condition |
| **P8** | M | 40-50 | Cardiology | Previous history of cardiac arrest and genetic cause suspected but unconfirmed; daughter being followed due to other cardiac condition but thus far no sign of same condition as father |
| **P9** | M | 70-80 | Cardiology | Previous history of cardiac problems, with a suspected genetic cause; family history of sudden cardiac death on father’s side |
